# A historical review of performance appraisal of public hospitals in China from the perspective of historical institutionalism

**DOI:** 10.3389/fpubh.2022.1009780

**Published:** 2022-10-10

**Authors:** Yaqing Li, Wensheng He, Li Yang, Keshuang Zheng

**Affiliations:** School of Management, Lanzhou University, Lanzhou, China

**Keywords:** public hospital, performance appraisal, historical review, driving force, framework of historical institutionalism

## Abstract

The government performance appraisal of public hospitals serves as an effective management tool to promote high-quality development. It is also an important means of realizing the “Healthy China” initiative. Since the founding of the People's Republic of China, changes in performance appraisal have been divided into the following four periods: the early stage of performance appraisal (1949–1977), the exploration period (1978–2008), the development period (2009–2018), and the integration period (2019–present). This clarifies the regional practice of public hospital performance and identifies the institutional factors of the evolution. It also demonstrates that administrative forces, market-driving forces, and mission-driving forces combine to bring about change in the public hospital performance appraisal system. However, problems such as insufficient application of performance appraisal and coordination between health administrative departments and medical insurance departments still loom large.

## Introduction

Health is a fundamental aspect of civil liberties and the necessary foundation for national development. Effective public health services are a powerful guarantee of public health (1). As an important supply force for the implementation of the “Healthy China” initiative, public hospitals not only undertake the major responsibility of providing medical services but also support the development of China's entire medical service system.

Public hospitals in China are the main body of the medical service system, which refers to the non-profit medical institutions sponsored by the government at all levels and included in the administration of government budgets at all levels, excluding primary medical and health institutions (clinics, health centers) and specialized public health facilities. They are operated to serve the public interests of the society. The government supervises public hospitals from the dimensions of access qualification, service price, quality, service behavior and personnel appointment The continuous advancement of public hospital reforms means that public management must deal with how to optimize the government's policies and measures for the performance management of public hospitals as a matter of urgency. There is a need to improve overall performance and to enable better adaptation of public hospitals as part of the construction and development of Chinese new-era socialism.

Performance refers to the effective and measurable consequences of an organization or individual through behavior based on certain organizational goals (2). It also involves efficacy, efficiency, or effectiveness. A hospital has a mission to fulfill social responsibilities and obligations, provide specific healthcare, and solve the health problems of patients in the most efficient and reliable way. Public hospital performance refers to the output and performance of specific organizational goals, such as effective use of medical resources, customer orientation, and responsibility (3). It also involves measuring the degree to which the mission of the hospital has been fulfilled. This includes factors such as clinical effectiveness, productivity, personnel, social responsibility, safety, and satisfaction (4). In the new public management movement, performance management was introduced into the public sector, with the core goal of improving public performance (5). The performance management model is designed for instrumental rationality and value rationality. It is a process whereby the public sector aims at 3E-efficiency, profit, and fairness to maximize public output (6). As a public organization oriented toward public welfare, public hospital performance management suggests a process in which hospital management identifies areas that require performance improvement, systematically implements performance improvement projects, sets goals, and continuously tracks indicators (7). The performance management of public hospitals in China can be divided into internal performance management and external performance management. Internal performance management means comprehensively managing the performance of teams and individuals within the organization and formulating reasonable and accurate goals to reinforce work efficiency and effectiveness (8). External performance management can also be categorized into administrative action by the government to supervise, manage, and guide the performance of public hospitals through goal setting, assessment, and result application, to enable public hospitals to better provide public medical services and continuously improve medical quality, safety, and healthcare. Therefore, features of welfare, fairness, and functional positioning should be highlighted. The performance appraisal mentioned in this article is a major link in the hospital performance management system (planning–execution–measurement–evaluation–feedback). It also includes the external performance assessment and evaluation of public hospitals carried out by the higher-level health administrative department. The related government department establishes an evaluation index system and regularly evaluates the organizational performance of public hospitals (9).

This paper identifies the changes in the performance appraisal of public hospitals in China since 1945 to explore the development stages, characteristics, and driving factors of these changes. Based on the analytical framework of historical institutionalism, this paper concentrates on the changes in the relationship between the government and hospitals in the reforms of the medical and health system and the financial compensation mechanism. The development stages of the evaluation system are identified, and the characteristics of each stage are summarized. This paper is also devoted to exploring the driving forces that lead to institutional change. This paper clarifies the changes in the driving forces during each period of change to analyze the dynamic factors, effects, and problems of public hospital performance management to better improve the efficiency of governance and to provide historical experience and advice.

## Sections on assessment of policy and implications

### The analytical framework of historical institutionalism

Thelen first clarified the concept of “historical institutionalism” in 1992 (10), taking research of the system as the content, and mainly focusing on how the system is formed by social change, how the system constrains individual behavior, and how the system interacts with the individual. From the perspective of historical institutionalism, history means more than a collection of a static series of events. It is a dynamic process (11). The basic approach of historical institutionalism to interpreting the influencing factors of institutional change includes two main aspects. The first one is the analysis of historical change, which taps into the influence of the economic system, political system, social concept, and other aspects of institutional change from the macro perspective. The history of previous Chinese public hospital performance appraisal policy reveals that its evolution can be divided into several stages featuring the policy characteristics of each stage. The second one is the analysis of the dynamic mechanism, which can be achieved by revealing the internal driving force of institutional change. This involves the analysis of the competition between different action subjects competing for scarce resources, resulting in the emergence of power asymmetry in the process of institutional evolution (12).

Combining the theoretical content with analysis of levels, historical institutionalism aims to explore the characteristics of each period with an emphasis on the “important nodes” of “quantitative changes which in history will lead to qualitative changes” (13). This research paper theoretically adopts historical institutionalism to explore the historical changes and evolution of Chinese public hospital performance appraisal policy. It constructs an analytical framework for public hospital performance appraisal policies. This paper focuses on path dependence and key nodes to present the transition of public hospital performance appraisal policy and analyzes the evolution logic of the background of the country's overall system and the interactions between aspects of the main body of the system (14).

### Changes in the performance appraisal of public hospitals in China since the founding of the people republic of China

In general, the system maintains a certain degree of consistency and stability. When the external environment undergoes major changes, the forces of various elements are intertwined, so that the system undergoes fundamental changes at a certain moment. Known as the “key node” of strong power (15), the choice of subject has a greater impact on the results in historical events (16). The performance appraisal policy of China's public hospitals has been severely affected by four external environmental changes, resulting in changes in the emphasis and characteristics of the policy.

**Figure 1:**
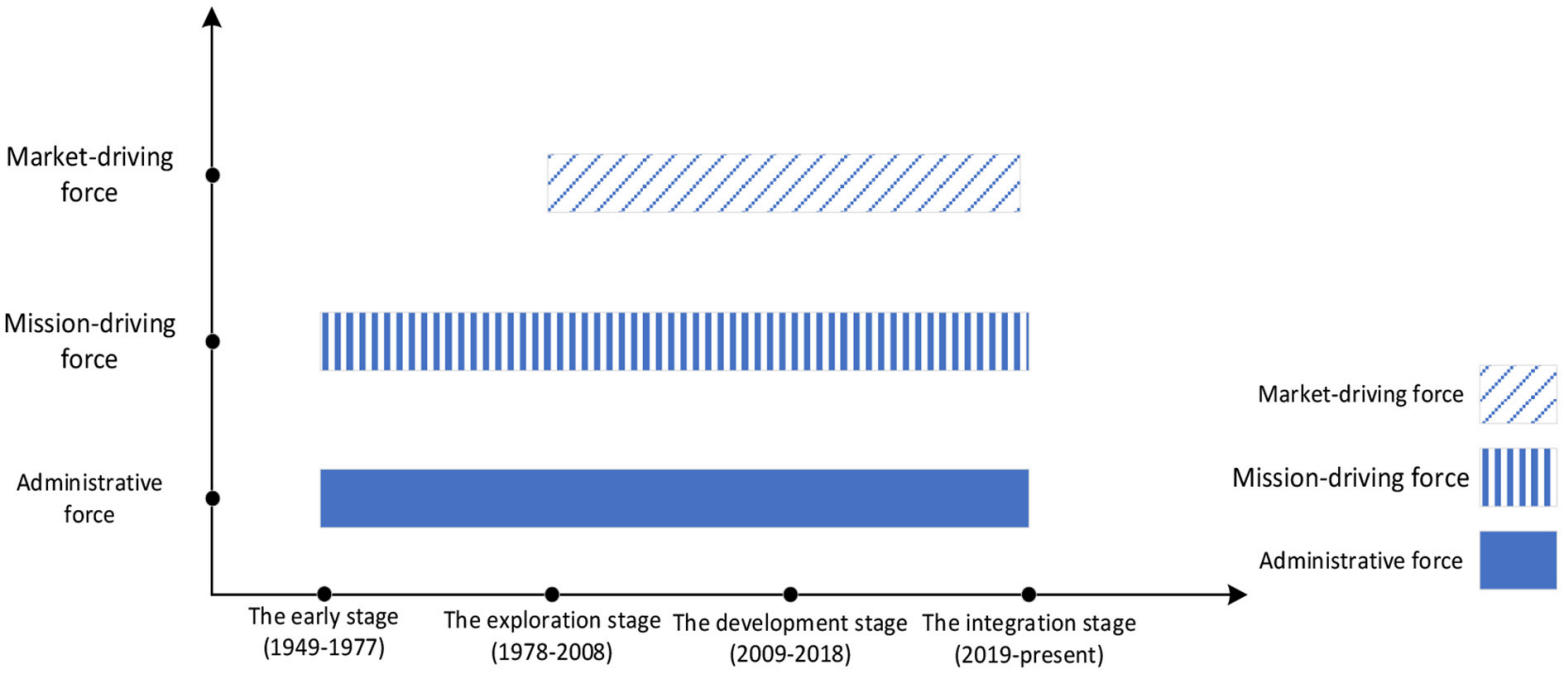
Analysis framework of historical changes in performance appraisal of public hospitals in China.

After the founding of the People's Republic of China, performance appraisal was not included in the national policies related to public hospital management until the “Opinions on Strengthening the Pilot Work of Hospital Economic Management” was issued in 1979. At that point, the state began to gradually loosen the operational autonomy of public hospitals in the form of a pilot program and through the management of hospital indicators. Therefore, public hospitals began to explore the market in the context of the market economy system, and performance appraisal gradually emerged. The publication of the “Opinions on Deepening the Reform of the Medical and Health System” in 2009 marked China's entry into a new round of medical and health system reform with the goal of making major changes to performance appraisal. In 2019, the “Opinions on Strengthening Performance Appraisal in Tertiary Public Hospitals” was released, signifying the launch of a national large-scale performance appraisal of public hospitals in an unprecedented policy measure. The government further promoted the development of public hospital performance assessment to verify the benchmarks for the overall performance of public hospital reform. Based on the key nodes in different historical stages, this paper divides the changes in the performance appraisal of public hospitals by the Chinese government into four stages since 1945: the early stage of performance appraisal (1949–1977), the exploration period (1978–2008), the development period (2009–2018), and the integration period (2019–present) (Figure 1).

#### The early stage of performance appraisal formation (1949–1977)

After the founding of the People Republic of China, the central government issued four guidelines for medical and health services to be oriented toward workers, peasants, and soldiers, integrating traditional Chinese and Western medicine and combining health work with mass movements (17). The overall goals of national medical and health services tend to be public health prevention. In addition to the medical treatment tasks, public hospitals at all levels also undertake some public health service tasks. Compared with public health prevention institutions, the government's management of public medical institutions at this stage mainly adopted economic means such as financial subsidies and drug price regulation. Public hospitals, as public institutions, are responsible for implementing plans and completing tasks (18). At this stage, the government had not yet formed the concept and operation mechanism of performance appraisal and performance management. In the policy documents for public hospital management, the government did not mention the keyword “public hospital performance,” which would be considered the threshold of the early stage of performance appraisal formation.

#### The exploration stage (1978–2008)

In 1979, the completion of the hospital's tasks should be evaluated from the dimensions of medical treatment, prevention, teaching, scientific research, and medical service quality are proposed. Through these dimensions, assessment indicators gradually took shape (19). In 1981, dpublic hospitals should set goals for tasks, hospital beds, establishment quotas, business technical indicators, and funding subsidies under the guidance of government plans. The word “task” mainly refers to confirming work efficiency and quality indicators in terms of scale, volume of work, and technical ability. Since 1985, the central aim of Chinese medical reform during this period was to “decentralize power and transfer benefits, expand hospital autonomy, and improve hospital efficiency,” and the orientation of hospital assessment indicators gradually shifted to efficiency. In 1992, the State Council's “Several Opinions on Deepening Health Reform” further expanded the autonomy of medical and health primary units, meaning that public hospitals had embarked on market-oriented reform (20). Since then, the government has stepped up the initiative of exploring the performance evaluation of public hospitals. In 2005, the hospital evaluation starts with “medical quality” and “satisfaction indicators” and moves toward medical quality and safety reviews. In this period, a comprehensive assessment was presented, including the assessment of medical quality, safety, and hospital operation management. The wave of new public management in the West, along with the trend of building a socialist market economy in reform and opening up, gradually affected performance management and the reviews of government and public organizations, whereby the form and content of assessment increased significantly (21).

#### The development stage (2009–2018)

In 2009, the industry, other social organizations, and individuals were encouraged to independently evaluate and supervise the performance of medical institutions. In 2010, the “Guiding Opinions on the Pilot Reform of Public Hospitals” proposed improving the supervision mechanism and performance evaluation system of public hospitals and strengthening the supervision of medical safety quality and economic operation. In 2015, the “Guiding Opinions on Strengthening the Performance Evaluation of Public Medical and Health Institutions” formally clarified the goals, principles, index systems, and evaluation standards of public hospital performance evaluation. Since 2016, the transformation of the evaluation of medical institutions from government-led to third-party evaluation are promoted gradually. It required that the evaluation should cover a range of factors, including social benefits, service provision, quality, safety, comprehensive management, sustainable development, and other indicators, as a way of establishing a hospital evaluation mechanism with quality as the core and public welfare as the orientation. In 2017, a public welfare-oriented assessment and evaluation mechanism were proposed to be established, the evaluation results of which would impact financial subsidies, medical insurance payments, and director's salaries. In 2018, the performance evaluation index based on patient satisfaction. The public health institutions at all levels to implement comprehensive performance appraisals were required. Overall, during the development period of performance appraisal, the government introduced policies related to the performance appraisal of public hospitals, signifying that performance appraisal had entered the exploratory stage.

#### The integration period (2019–Present)

In 2019, the “Opinions on Strengthening the Performance Evaluation of Tertiary Public Hospitals” were issued. The National Health Commission and other departments conducted performance evaluations on 2,398 tertiary public hospitals across the whole nation, which is called The “National Examination Model for Public Hospitals.” It consisted of 55 three-level indicators in four dimensions: quality, operation, sustainable development, and patient satisfaction. The national performance appraisal attaches great importance to the application of the appraisal results. In response to data quality control issues, the National Health Commission urged all hospitals to complete assessments and make the necessary changes within a time limit. In 2020, the “proportion of revenue from high-value medical consumables” was incorporated into the evaluation index system of tertiary public hospitals. In the same year, second-level public hospitals started to carry out the performance evaluation (Table 1).

**Table 1:** Historical changes in performance assessment of public hospitals.

**Stage**	**Content**	**Goal**	**Evaluation**	**Application of evaluation result**
The early stage (1949–1977)	Medical quality, public health prevention	To improve social welfare based on public health prevention	Unified revenue and expenditure, fixed tasks	Almost none
The exploration period (1978–2008)	Medical quality, patients' satisfaction, economic profits as a major concern indicator	To continue to meet the growing medical and health demands of the people	Comprehensive assessment such as hospital management evaluation	Over-emphasis on economic impulse
The development period (2009–2018)	Social benefits, service provision, quality and safety, comprehensive management, sustainable development	To follow the principles of public welfare, and social benefits, and adhere to the patient-oriented approach	From local government assessments to third-party independent evaluations	The assessment results are linked to the financial subsidies of public hospitals, personal income, appointment and dismissal of the director, hospital grade review, etc.
The integration period (2019–present)	Quality, management, sustainability, satisfaction, Health emergency	To adhere to the principle of the people's health as the center and promote the high-quality development of medical services	From local government assessment to national assessment	The assessment results are linked to the financial subsidies of public hospitals, personal income, appointment and dismissal of the director, hospital grade review, etc.

The national health administrative department plays the role of “benchmarking” through the “national examination” to guide the positioning and development of public hospitals in medical reform. At the same time, the results of performance assessment are made public through public hospital rankings to promote public supervision. Simultaneously, the provincial government is engaged in the performance appraisal of regional public hospitals and continuously strengthens the application of the results. According to the actual situation of the locality, combined with the national performance indicators, more scientific and reasonable performance appraisal indicators are added. Then, the review can be more in line with the actual situation of the region, and effective performance incentive models within different levels, attributes, and disciplinary characteristics can be implemented. Therefore, public hospitals benchmark internal weak links through national monitoring indicators, and constantly revise and adjust internal performance management.

### Regional practice of performance appraisal in public hospitals

The term “public hospital performance” first appeared in policy texts in 2004, and it appeared more frequently in 2009. The concept of performance appraisal has been widely applied in the management of public hospitals. Terms used in relation to the government's supervision of public hospitals are “assessment,” “management,” “rewards and punishments,” and “indicators.” In other words, the use of index assessment, reward, and punishment measures to supervise public hospitals implies that government supervision already existed before formal performance appraisal. China already had a practical foundation in these aspects. The main indicators of the government's assessment of hospitals focus on two aspects: quality and efficiency. Since 2009, important indicators such as “service” and “sustainable development” have gradually been added.

With the deepening of the new round of medical reform, various regions are exploring public hospital reform while different models of performance appraisal have emerged in each region, including the performance appraisal of hospital directors and local governments. The performance appraisal of local public hospitals typically uses the following models: the Shanghai Shenkang model, the Beijing Hospital Administration model, the Fujian Sanming model. The accumulation of regional performance assessment experience and improvements in information systematization, based on local performance assessment experience and national policy orientation, led to the launch of the national performance assessment in With the deepening of the new round of medical reform, various regions are exploring public hospital reform while different models of performance appraisal have emerged in each region, including the performance appraisal of hospital directors and local governments. The performance appraisal of local public hospitals typically uses the following models: the Shanghai Shenkang model, the Beijing Hospital Administration model, the Fujian Sanming model. The accumulation of regional performance assessment experience and improvements in information systematization, based on local performance assessment experience and national policy orientation, led to the launch of the national performance assessment in 2019. This means that the performance assessment of public hospitals has moved toward the implementation of national standards. Performance appraisal of the Shanghai Shenkang model.

1. Shanghai Shenkang Model

The Shanghai Shenkang model, in conjunction with the Suzhou, Beijing, and Wuxi models, was launched to pilot the innovative model of “separation of management and administration” in China's medical reform (22). The Shanghai Shenkang Hospital Development Center is a non-profit public legal entity enterprise established by the municipal government. Since 2006, it has taken the lead in implementing the annual performance appraisal of public hospital directors in China, and applied for many years to carry out annual performance appraisals. The 23 five-dimension appraisal indicators, ranging from social satisfaction rate to effective management, asset management, sustainable development, and employee satisfaction, are used to quantitatively assess operational performance. In addition, qualitative indicators such as safe construction and hospital-running direction are used to assess the operational performance and the management performance of directors of municipal hospitals. From the perspective of the application of performance appraisal results, the combination of annual appraisal and tenure appraisal, the unification of results appraisal and process appraisal, and the association of results with rewards and punishments are powerful institutional constraints.

The performance appraisal of directors is also a part of the appraisal system. Meanwhile, the management of hospital directors serves as a management tool to promote hospital performance (23). The results of performance appraisal are directly used as an important basis for the director's annual performance rewards and punishments, selection and appointment, evaluation of excellence, and as an important basis for the approval of the hospital's total salary budget (24). The Shenkang Model has established a set of hospital performance appraisal and evaluation mechanisms with public welfare as the core element in operational efficiency. Therefore, it has realized the organic unity of the external and internal performance appraisal of the hospital. The level of refined management of public hospitals can be promoted from the dimensions of medical expenses, hospital scale, work efficiency, medical cost control, and scientific research level (25).

2. The Beijing Hospital administration model

The Beijing Municipal Hospital Administration Bureau was established on July 8, 2011. Since 2012, the Beijing Municipal Hospital Administration has conducted performance evaluations on 22 municipal tertiary public hospitals (26). It has established a public hospital evaluation system with public welfare as the core standard. It has unified the four dimensions of social evaluation, internal management, operation efficiency, and development strength to achieve a combination of quantitative and qualitative evaluation. In the model, 25 assessment indicators have been devised to conduct an assessment of the hospital's management direction, operational efficiency, the hospital's sustainable development, and talent team building (27). It has also established a public system of performance assessment ranking and indexing, which contributes to decisions on the appointment and dismissal of the hospital director, and performance-based annual salary. The characteristics of the Beijing Hospital Administration model are as follows: First, patient satisfaction, accounting for 10% of the review results, highlights public welfare performance. Second, it strengthens the application of performance appraisal results, adopts integrated assessment, and associates performance assessment results with the appointment and dismissal and the evaluation of the merits of administrative directors. Beijing Municipal Finance issued special monetary rewards for performance appraisal results, the value of which amounted to one billion yuan by 2018.

3. The Fujian Sanming model

As pioneers in medical reform, Sanming City of Fujian Province have implemented several medical reform policies, which are foremost at a national level. After the government increases financial support, the performance assessment of hospitals has to be strengthened to enhance efficiency and eliminate resource waste. The directors of the Sanming public hospitals are evaluated on 40 items in six categories, including directing the running of a hospital, hospital management, hospital development, and service evaluation. There are quantitative standards for each item, especially the proportion of drug income to total income, average cost, and average length of hospitalization, all of which are strictly controlled. An annual salary system is implemented for the directors of Sanming City Hospital. The Sanming Medical Reform Leading Group conducts a comprehensive assessment annually of the performance of the director, and the results of the assessment serve as an important basis for the director's annual salary, selection and appointment, management supervision, incentives, and constraints.

Sanming City has effectively strengthened the supervision of medical services, tightened the management of drug use in medical institutions, and standardized the list of centralized procurement of drugs by public hospitals. The government closely monitors the management of hospitals, the increase in the average cost of outpatient and inpatient visits, and the use of antibacterial drugs and auxiliary drugs. Unauthorized drug use and other illegal behavior are notified to the authorities. Fujian province has strengthened the effective connection between the performance evaluation of tertiary public hospitals and the comprehensive reform effect of public hospitals, thereby increasing the overall application of the evaluation results.

In the past, before the introduction of the national tertiary public hospital performance appraisal policy, the performance appraisal of public hospitals and departments shared the characteristics of “assessment introversion.” Except the regional practice of performance appraisal of China's public hospital, there is another way to evaluate the public hospital performance, the evaluation from the medical insurance department. At present, there are three sources of financing for public hospitals in China. They are as follows: public financial investment represented by financial subsidies, social medical security represented by medical insurance funds, and personal payment represented by citizens' payments. Currently, financial investment in public hospitals accounts for a relatively low proportion of the overall income of public hospitals, and payments from medical insurance funds have become a key part of their income. Therefore, the sources of the overall financing and compensation of public hospitals are mainly derived from medical insurance payment funds.

Medical security departments at all levels, as public payers, have established a “public contract” model with public hospitals to supervise and assess them. Unlike performance appraisal under administrative governance, which is directly under the command of the health administrative department, the National Medical Security Administration has become a key department, going beyond the Health Commission with substantial appraisal incentives for and constraints on public hospitals. The assessment of key indicators enables the medical security department to manage the inspection fees and the drug consumables expenses of public hospitals, thereby regulating medical service behavior and improving the quality of services. The assessment results are linked to the refund of security deposits, the renewal of medical insurance service agreements, and the total budget indicators for the next year (28). Furthermore, the application of assessment results is directly related to the income of public hospitals' medical security funds and the income of the medical staff. As a result, the performance assessment of medical security departments has been given more and more attention by public hospitals in recent years (29).

### Institutional factors and driving forces affecting the performance evaluation of public hospitals

North believes that institutional analysis is concerned with history (30). Institutional analysis should focus on the internal logic of institutional change (31). The evolution of any policy needs to follow certain rules, and the performance appraisal of public hospitals is no exception. In addition to the influence of macro-structural factors, the evolution of the performance appraisal policy of public hospitals and the power to provide a strong internal driving force for the reform of performance appraisal policy are important factors. Since the founding of the People's Republic of China, the transformation of the main social contradictions, the changes in the health needs of citizens, and the evolution of public hospital performance assessment have always been inseparable from the gradual progress of the national administrative system and the medical and health system. It is also inextricably linked to the increasingly efficient market economy system and the rapid development of medical and health technologies. With this in mind, this paper divides the influencing factors that promote change in public hospital performance appraisal into three forces: administrative-driving force, market-driving force, and mission-driving force.

1. Institutional factors

(1) Changes in the reform of the economic system

The changes in the performance appraisal of public hospitals in China provide a glimpse of the changes in the reform of the economic system. The founding of People's Republic of China has seen a transformation from a planned economy to a socialist market economy, which determines the basic policy orientation for the supply and demand of medical services. The market-oriented economic system is more inclined to adjust the medical service market through market mechanisms. This results in reduced government intervention in medical supply and demand. Under all socioeconomic systems, public hospitals perform a variety of socioeconomic functions, and there may inevitably be conflicts between these functions (32). Public hospital managers set specific tasks and objectives in a specific time frame. The tasks and objectives are often divided into two categories: economic tasks and public welfare tasks. The latter are regarded as a social function, generally including the provision of public goods with positive external properties (medical education and practice, medical research and development, epidemiological detection, infectious disease prevention, public health education, etc.) (33). The provision of low-cost medical services and other health services for the poor are also included. Reducing excessive medical care also curbs the rapid growth of health care costs.

(2) Changes in the mechanism of financial investment in health

As the goals of economic development progress through different stages, the financing system of public hospitals undergoes essential changes. Government performance goals are similarly transformed. As a precious source of medical and health development, finance also serves as the foundation of national governance. The mechanism of medical financial investment plays a pivotal role in improving the performance of public hospitals. From 1949 to 1978, administrative planning and directives were the priorities. The medical and health services were funded centrally by the government, and the personnel were also supported by the government. the public hospitals were managed through “unifying revenue and expenditure.” Under the system, all income had to be turned over, and expenditure was ratified in an annual budget calculated by the competent department. There might have been differences in the form of “fixed subsidy” and “differential subsidy.” From 1979 to 2009, the autonomy of hospital operations gradually increased, and financial subsidies continued to decrease. In 1979, the state adopted a “full-scale management, quota subsidy, and balance retention” policy for public hospitals. This is the origin of the fixed subsidy for public hospital beds. In 1988, in terms of setting up tasks, establishing quotas, and quality tasks with the health authorities, public hospitals are entitled to greater autonomy. Medical staff are also encouraged to take part-time jobs as a way of enhancing staff incentives. Since 2009, The government at all levels was obliged to establish a government-led, multi-dimensional health investment mechanism. Government health input should take into account both supply and demand sides, and the central and local governments should assume responsibility for health financial input at different levels (34).

(3) Changes in the medical and health systems

As an important variable affecting the historical process of performance appraisal of public hospitals, changes in the medical and health system clearly demonstrate how the interaction between the government, the market, and society promotes the process and the alternate evolution between the fairness and efficiency of medical and health services. From 1949 to 1978, China established the basic medical and health system, characterized by collective egalitarianism with an emphasis on the fairness of medical and health services. Therefore, the “barefoot doctor” system and the cooperative medical system were prevalent. From 1979 to 2009, the basic medical and health system established in the previous stage was affected by the market-oriented economic system, which tended to improve the efficiency of medical services and the development of the supply side, to the detriment of fairness. Since 2009, the new round of medical and health system reform has clarified the development direction of fairness and public welfare, with the medical and health system gradually transforming from efficiency to fairness. Public hospitals certainly serve as the main objects of the reform of the medical and health system. This is evident in the identification of the hospitals' public responsibilities and the setting of management goals by central and local governments. The evolution from the absence of special performance appraisals to the introduction of performance appraisals and performance management goals reflects the changing goals of the healthcare system.

The changes in the performance appraisal of public hospitals over more than 70 years reveal the development of public service tasks of public hospitals, from prevention and control to treatment, to a prevention-based approach, a combination of prevention and treatment. The characteristics of performance appraisal have evolved from a focus on public welfare, a reduced focus on public welfare, a return to a focus on public welfare, the maintenance of public welfare, and the application of performance appraisal results. The performance objectives have gradually moved from “treatment-centered” to “health-centered,” and the content of performance appraisal developed from the initial medical quality and public health services to medical quality and operational efficiency, sustainable development, patient satisfaction, and other multi-dimensional indicators.

(4) Changes in medical and health needs

The historical review of the performance appraisal process shows that the performance objectives of public hospital performance assessment have changed in relation to the level of social medical services and the health status of patients.

The main tasks of public hospitals range from prevention and control to treatment, to prevention first, and to a combination of prevention and treatment. Therefore, the core goals evolve from promoting public welfare, weakening public welfare, returning to public welfare, and maintaining public welfare. Public welfare refers to providing medical services to the public based on the value of public medical services on the premise that the government holds the main responsibility and financial investment, whether it is “patient-centered” or “people's health-centered.” In short, public hospitals are for the people, and the government entrusts public hospitals with the provision of public medical services.

2. Driving force

(1) Mission-driving force

The term “mission” refers to the position, role, and obligation of an organization in social progress and social and economic development (35). The term explains the fundamental nature and meaning of an organization (36). Mission-driving forces are responsible for the achievement of an organization's mission. The mission determines the ultimate goal of the organization's input and output. Therefore, the formulation of any organization's performance appraisal goals or programs must be closely related to its mission (37). The mission of public hospitals is to provide medical and public health services, complete the task of cultivating skilled workers at the clinical stage, and succeed in clinical research (38). Ultimately, it is the standard and significance of the government's performance appraisal and management of public hospitals. Therefore, the mission-driving force of the changes in the performance appraisal of public hospitals in China is inseparable from that of public hospitals, prompting the government to continuously promote this assessment so that public hospitals can clarify their functional positioning, deliberate on their responsibilities, and improve their performance, ensuring that people have access to public medical services.

(2) Administrative forces

“Administration” is the term used for the execution of the will of the state (39), whereby there is an ordered hierarchical system, running from top to bottom, and accepting the administrative control, and guidance of the government (40). Administrative force responds to public and controlled administrative means or methods such as national policies and regulations. Policies and administrative orders in the country's overall strategic planning, medical, and health system reform represent the administrative force for changes in the performance assessment of public hospitals.

In the early stage the national health level needed to be improved urgently. Under these circumstances, the government regarded medical and health services as social welfare and established a basic medical and health system, whose policies tended to focus on the fairness of medical and health services (41). In 1950, under the planned economy system, government financial subsidies could cover more medical expenses (42). The medical and health services, and the personnel, were fully funded and supported by government finance. Meanwhile, all aspects of medicine are subject to comprehensive state control. The government's management of public hospitals is dominated by administrative planning instructions (33). Public hospitals, at all levels, undertake significant public health functions such as vaccination, health awareness programs, and education on infectious diseases. During this period, public organizations, including public hospitals, had a strong mission-driving force. In the early stage of performance appraisal formation, public hospitals needed to fulfill their responsibilities. Although there was no clear government performance appraisal system, the government's performance management for hospitals came into existence in the form of “unified planning, unified configuration, and unified management” (43), as represented by administrative and mission-driving forces.

As public hospitals compensate for the lack of financial investment in health care by means of promoting economic benefits for hospitals with high profits from drugs, the phenomenon of excessive medical services is becoming frequent, and conflicts between doctors and patients frequently occur. Given these conditions, the “2005 Human Development Report,” issued by the United Nations Development Program Office in China concluded that “China's medical reform is basically unsuccessful, and the medical reform deviates from the public welfare of the hospital” (44). In 2009, a new round of medical reform was launched, and the medical and health system gradually shifted from efficiency to fairness (45). The transition from public hospitals to public welfare was continuously strengthened, and mission-driving emphasis was placed on public welfare indicators. The government's financial investment in public medical care remains relatively low, while the scale of profit growth in public hospitals is controlled, emphasizing the quality of development. The government's leadership, security, management, and supervision responsibilities for public hospitals are being consistently enhanced. The indicators are constantly changing. Excessive medical treatment is suppressed by cost control indicators, while full consideration is given to the quality of indicators.

(3) Market-driving Force

The term “market” conveys the assumption of economic man, of a series of rules and norms formed by various stakeholders in the market through free competition that shapes the behavior and cognition of enterprises (46). The market-driving force responds to the market through self-interest and profit (47). The market-driving force of the changes in the performance appraisal of public hospitals emerges from the market law that public hospitals provide public medical services as the main entity of the market under the development of the market economic system (48).

Since 1979, China has gradually made the transition to a market economy system. The government's continuous investment in hospitals has led to increasing pressure on government expenditure. Since the reform of the government's fiscal and taxation system, the proportion of government health expenditures to total expenditures has continued to decline. In principle, government health input should not be lower than the growth rate in fiscal expenditure (49). However, in the market economy system, enterprises became the pioneers of reform and opening up. Enterprises began to make profits under the market economy. Hospitals as institutions experienced the natural drive to generate more income, as did medical practitioners. Coupled with the increasing pressure of financial investment, the government and the hospital's exploration of the market economy system can be described as a perfect match. The 1985 “Report on Several Policy Issues in the Reform of Health Work” marked the real beginning of work on China's medical reform (50). In 1988, in terms of self-management and the self-control of financial revenues and expenditure, public hospitals can be given greater autonomy to set tasks, and establish quotas and quality standards by the governing authorities. This relaxation should include gradually loosening control over drug prices and purchases. Hospitals are allowed to set prices and formulate drug procurement plans independently (51). In so doing, hospitals are generating more income, and they are operating better than before, based on increased market impulse and reduced government input. The government seems to have loosened regulations. However, the government's control is not weakened in terms of tight control of establishment quotas, salaries, professional titles, and other aspects. Only the compensation mechanism has changed (52). From the perspective of contradictions in social development, the demand for medical and health services is constantly increasing, while the planned allocation of resources is limited. However, the impact on assessments of emphasizing efficiency is that hospital assessment emphasizes economic benefit indicators, overusing quantitative indicators, and the application of evaluation results overemphasizes the economic aspects. Market forces are gradually playing a role, while mission-driving forces seem less effective, and there is not much emphasis on indicators such as functional positioning. At this stage, market-driving forces gradually play a role, taking a dominant position while the administrative force, while still in play, is weaker than at the previous stage, and the role of the mission-driving force is weakened.

Since 2019 and the implementation of the “Healthy China” initiative, the state has successively issued policies related to public hospitals, and performance appraisal of national tertiary public hospitals shows that performance appraisal has entered a period of integration. Since the COVID-19 pandemic, the assessment began to focus on public health emergencies, such as major epidemics. Simultaneously, China vigorously promoted a strong anti-epidemic spirit and a highly professional spirit. The mission-driving forces promoted the continuous enhancement of the quality performance of public hospitals. In the context of improvements in medical and health technology and the continuous increase in citizens' health needs in the post-epidemic era, the market-driving force promotes the continuous improvement of the quality and operational performance of public hospitals. In 2021, the administrative force still prevails, and the indicators of quality and safety, operational efficiency, sustainable development, and satisfaction now have general attention. The performance appraisal system of public hospitals has entered a new stage of performance appraisal in mission-driving, market-driving, and administrative-driving collaborative governance.

3. Summary

The paper identifies and analyzes the impact of the three driving forces on the performance assessment of public hospitals in the process of historical changes. Based on the national system and the public welfare attributes of public hospitals, at each stage of the development of public hospital assessment in China, the administrative force has always come through as an externally born force leading the development trend of performance assessment. The market-driving force began to emerge during the evaluation period (1978–2008), constantly improving the performance appraisal system. The mission-driving force is an internal force, generated by the interaction between the professional characteristics of the medical service and the external social environment. Therefore, there may be conflicts or collaboration among these three factors.

## Actionable recommendations

In China, public hospitals are aspects of medical and health service supply, as well as being the leading force in the development of medical and health services and medical science and technology. Rapid changes in diseases, the frequent occurrence of public health emergencies, and the urgent public demand for medical services and health are the key driving forces for the high-quality development of public hospitals in their drive to improve the organizational performance of public hospitals. Therefore, promoting the performance appraisal of public hospitals as an effective means of administrative management is inseparable from the deepening of the application of appraisal results and the coordination among the main departments of appraisal.

Local governments should continue to strengthen the application of the results of performance appraisals. They should set more reasonable indicators of performance appraisal that are more in line with regional performance appraisals, and effective innovation performance incentives at different levels, attributes, and disciplines depending on local conditions and national standards. Among government departments, the performance assessment and management of public hospitals should be sufficiently coordinated. The performance appraisal of the Health Commission should be intensified at all levels to supervise the medical quality, medical safety, and medical services of public hospitals. Similarly, cooperation with medical insurance departments at all levels should be standardized to regulate medical service behavior and control medical expenses through assessment.

In the post-epidemic era, the continuous integration of information technology and technology-driven medical enterprises should be strengthened. The real-time sharing of data, such as personal health codes in a manner that has become normalized for epidemic prevention and control, online appointments, the quality of electronic medical records, medical insurance data informatization, and overall performance appraisal data, would be improvements. The collection of national, local, and public hospital performance appraisal data connection should also be included, thereby indicating that the technical support of future public hospital performance appraisal is the key element for public hospital performance improvement and the technical guarantee for the effective implementation of public hospital performance appraisal.

## Conclusions

### The effectiveness of performance appraisal

As a result of the changes in and evolution of public hospital performance assessment, the exploration of the local public hospital performance assessment experience has embraced the flourishing of the Shanghai Shenkang model, the Beijing Hospital Administration model, the Fujian Sanming model, and other local government assessment models for public hospitals. The implementation of the national tertiary public hospital performance appraisal policy will gradually achieve the dual goals of “external responsibility” and “internal control” in the performance appraisal of public medical institutions. In the process, the “national examination” plays the role of benchmarking helping the positioning and development of public hospitals in medical reform. For example, the publication of public rankings reinforces public supervision. As an important source of performance appraisal's effectiveness, the data show that the appraisal indicators are becoming clearer, content is more and more refined, and the methods used are more comprehensive and intelligent. Meanwhile, as a result of performance appraisal, the quality of medical services and management levels have continued to improve, functional positioning has been implemented, medical quality and safety have been enhanced, hospital management and internal management have been strengthened, a sustainable development mechanism has been maintained, and patient satisfaction has been steadily promoted. The government is more inclined to determine the assessment objectives, with a focus on the assessment results. As a result, autonomy is handed over to public hospitals to enable hospitals to advance the ways of encouraging public hospitals to give full play to their initiative and elevate their performance in an all-round way.

In the first two stages, the government exercised excessive management, including control over recruitment, finances, and daily operation (53), affecting the autonomy of public hospitals and reducing the vitality and efficiency of hospitals. The promotion of the target assessment system has the inherent advantage of focusing on the target task. However, in relative terms, the target task setting is too broad to make progress. This is not conducive to the improvement of the overall performance of the hospital. The continuous improvement of the assessment system and the macro policy orientation for the high-quality development of public hospitals enables the government to use assessment as a management method. In this way, the government takes the leading responsibility by determining assessment objectives and focusing on assessment results. However, the autonomy of the operation and the management belong to the public hospitals, which are encouraged to give full play to their initiative and improve their performance in an all-round way.

### Existing problems

First, the application of performance appraisal results needs to be strengthened along with the feasibility and rationality of performance appraisal indicator settings. More efforts need to be made to make performance appraisal results more effective. At present, the application remains ambiguous, with large differences between regions, and there is unbalanced and insufficient development. In some regions, appraisal applications can use performance information effectively to set more reasonable performance goals, and reward-punishment systems based on performance appraisal.

Second, the collaboration between medical insurance and medical service departments on the performance appraisal of public hospitals needs further improvement. At present, the proportion of financial funds in the overall income of public hospitals remains relatively low. Therefore, the overall financing compensation incentives of public hospitals mainly depend on the payment of medical insurance funds (54). Medical security departments at all levels supervise and assess public hospitals with the “public contract” agreement model (55). Relying on the assessment of key indicators, the government takes control of the inspection fees and drug consumption to standardize medical behavior, improve the quality of medical services, assess results, and refund security deposits. The renewal of the medical insurance agreement and total budget index is closely related to assessment results. Therefore, medical appraisal processes in the context of public hospitals have attracted increasing interest in recent years.

## Author contributions

YL: substantial contributions to the conception or design of the work, or the acquisition, analysis, or interpretation of data for the work, and drafting the work or revising it critically for important intellectual content. WH: provide approval for publication of the content, agree to be accountable for all aspects of the work in ensuring that questions related to the accuracy or integrity of any part of the work are appropriately investigated, and resolved. LY and KZ: revising it critically for important intellectual content. All authors contributed to manuscript revision, read, and approved the submitted version.

## Funding

National Natural Science Foundation of China (71974087). Research on the Influence Mechanism of Fiscal capacity, departmental Game and organizational Culture on Performance Management Reform of Local government Budget in China. Social Science Planning Project of Gansu Province: Research on Remodeling and Upgrading of Business Environment in Gansu Province (19YB054); Gansu Province Service Local Social Development Project: Research on Business Environment Optimization in Gansu Province Based on Large-scale Data Survey and Analysis (2019-FWZX-09); 2000USD received for open access publication fees.

## Conflict of interest

The authors declare that the research was conducted in the absence of any commercial or financial relationships that could be construed as a potential conflict of interest.

## Publisher's note

All claims expressed in this article are solely those of the authors and do not necessarily represent those of their affiliated organizations, or those of the publisher, the editors and the reviewers. Any product that may be evaluated in this article, or claim that may be made by its manufacturer, is not guaranteed or endorsed by the publisher.
